# Effect of lncRNA WT1-AS regulating WT1 on oxidative stress injury and apoptosis of neurons in Alzheimer's disease via inhibition of the miR-375/SIX4 axis

**DOI:** 10.18632/aging.104079

**Published:** 2020-11-21

**Authors:** Quanbao Wang, Xiumin Ge, Jie Zhang, Licheng Chen

**Affiliations:** 1 Department of Neurology, The People’s Hospital of Linyi City, Linyi 276000, P.R. China; 2 Department of Neurology, Linyi Mental Health Center, Linyi 276000, P.R. China; 3 Department of Emergency Internal Medicine, The People’s Hospital of Linyi City, Linyi 276000, P.R. China

**Keywords:** lncRNA WT1-AS, WT1, miR-375, SIX4, Alzheimer's disease

## Abstract

Objective: To study the effect of lncRNA WT1-AS on oxidative stress injury (OSI) and apoptosis of neurons in Alzheimer's disease (AD) and its specific mechanisms related to the microRNA-375 (miR-375)/SIX4 axis and WT1 expression.

Results: After bioinformatic prediction, WT1-AS was found to be downregulated in Aβ_25-35_treated SH-SY5Y cells, and WT1-AS overexpression inhibited WT1 expression. WT1 could target miR-375 to promote its expression. miR-375 bound to SIX4, and miR-375 overexpression inhibited SIX4 expression. WT1-AS inhibited OSI and apoptosis, while WT1 and miR-375 overexpression or SIX4 silencing reversed the WT1-AS effect on OSI and apoptosis. *In vivo* experiments revealed that WT1-AS improved learning/memory abilities and inhibited OSI and apoptosis in AD mice.

Conclusion: Overexpression of WT1-AS can inhibit the miR-375/SIX4 axis, OSI and neuronal apoptosis in AD by inhibiting WT1 expression.

Methods: Related lncRNAs were identified, and miR-375 downstream targets were predicted. WT1-AS, WT1, miR-375 and SIX4 expression was detected in a cell model induced by Aβ_25-35_. The binding of WT1 with miR-375 and that of miR-375 with SIX4 were further confirmed. Adenosine triphosphate (ATP), reactive oxygen species (ROS), malondialdehyde (MDA), superoxide dismutase (SOD), glutathione peroxidase (GSH-Px) and lactate dehydrogenase (LDH) activities, and apoptosis levels were tested after mitochondrial membrane potential observation. Learning/memory abilities and neuronal apoptosis were tested in a mouse model.

## INTRODUCTION

Neurodegenerative disease is an umbrella term for a range of conditions that is associated with the loss of synapses between neurons in the brain [1]. One such disorder, Alzheimer's disease (AD), is recognized as the most common neurodegenerative disease in elderly individuals and is characterized by progressive cognitive impairment and memory loss [2, 3]. Aging has been identified as the foremost cause of neurodegenerative diseases, and the incidence of neurodegenerative diseases is expected to surge with an increase in aging populations over the next few decades [4]. In the clinical setting, AD is primarily manifested by progressive memory disorder, cognitive dysfunction, personality changes and language disorder, which has serious negative effects on the social wellbeing, work and life of elderly individuals and results in a heavy burden on the family and society [5, 6]. AD itself is a highly complex neurodegenerative disease involving multiple mechanisms, which has caused serious problems for the development of efficacious treatment regimens for this disease [7]. Recent decades have also witnessed a surge in investigations on mitochondrial defects and oxidative stress, such that an increasing number of studies support the notion that that mitochondrial defects and oxidative stress exert negative effects on the upstream pathological events of AD, which has paved the way in providing a theoretical basis for the early diagnosis and treatment of AD [8, 9].

The development of AD shows a certain relationship with age and lifestyle habits, such as smoking, high calorie intake, and lack of proper exercise, as well as medical history of hypertension, brain injury, diabetes, hypercholesterolemia, hyperhomocysteinemia, etc., all of which can lead to or aggravate oxidative stress. Theoretically, the elimination of oxygen free radicals can act as a protective factor in AD [10, 11]. Moreover, the main function of mitochondria is oxidative phosphorylation to produce adenosine triphosphate (ATP), which provides energy for maintaining the normal physiological functioning of cells, and mitochondria are also the key site of oxygen metabolism and reactive oxygen species (ROS) production *in vivo* [12]. In addition, mitochondria are generally known as the converging point of cell death pathways and the main site of oxygen free radical production, whereas mitochondrial dysfunction can also lead to various neurodegenerative diseases [13, 14]. Although the human brain accounts for only 2%~3% of the total body weight, oxygen consumption by brain tissue accounts for 20%~30% of total oxygen consumption given that neurons have virtually no energy reserves [15]. The energy used to maintain normal physiological function of neurons primarily comes from ATP produced by oxidative phosphorylation, and mitochondria are the processing plants of the required cell energy [16]. Therefore, mitochondrial dysfunction will inevitably lead to neuronal damage or even death [17]. Furthermore, neuronal death is a common feature in some neurodegenerative diseases [18]. Additionally, mitochondrial dysfunction has also been closely associated with neurodegenerative diseases, including AD [19, 20].

In 1992, Hardy and Higgins proposed the amyloid β (Aβ) theory, which has been the mainstream theory, that Aβ protein aggregation is the initiating factor for the pathological damage that is seen in AD patients [21]. The formation and aggregation of Aβ is also known to stimulate a cascade of cell events, such as mitochondrial dysfunction, Tau protein hyperphosphorylation, neuron apoptosis and synaptic degradation [22]. Furthermore, Aβ can enter human mitochondria, promote the production of ROS, and induce oxidative stress [23, 24]. Therefore, it is reasonable to suggest that Aβ can play a toxic role through oxidative stress and can also induce oxidative modification of various biological molecules *in vivo*, including lipid peroxidation of the cell membrane, peroxidation of lipoproteins, and oxidative modification of DNA and RNA, to ultimately exert irreversible damage to neurons, all of which can play a deciding role in the fate of AD [25, 26].

In addition to the above hypothesis, the role of long-chain noncoding RNAs (lncRNAs) and microRNAs (miRNAs) at the epigenetic, transcriptional and posttranscriptional levels to increase/decrease gene expression has been greatly explored in numerous studies, which have indicated their broad participation in various physiological and pathological processes. MiRNAs are a group of small noncoding endogenous and evolutionarily conserved posttranscriptional regulatory RNAs that are approximately 22 nucleotides in length [27]. LncRNAs are a type of noncoding RNA longer than 200 nucleotides with limited or no protein-coding potential and are involved in cell growth, proliferation and differentiation [28]. In addition, there seems to be a complex regulatory relationship between lncRNA and miRNA in the aspects of dose compensation effect, genomic imprinting, DNA methylation, histone modification, chromatin remodeling and many other epigenetic processes [29, 30]. Their interaction is involved in the occurrence and development of several diseases, including neurodegenerative diseases [31]. Another molecule of interest, the gene-encoding SIX homeobox 4 protein (SIX4), also known as AREC3, is a member of the homeobox gene subfamily of transcription factor genes, which not only possesses the ability to differentiate and develop embryonic cells but also induce cell proliferation, invasion and metastasis, exerting a significant function in the occurrence and development of tumors and other malignancies [32]. Moreover, SIX4 also participates in organ development, such as myogenesis, olfactory placode development and neurogenesis. [33, 34]. In addition, the transcription factor WT1 can encode four homologous isomers through selective splicing and has significant functions in transcription, splicing and the regulation of basic cell functions such as proliferation, differentiation and apoptosis [35, 36]. However, there are currently few systematic studies on lncRNA/miRNA/gene interactions in neurodegenerative diseases (e.g., AD).

Accordingly, the current study set out to investigate the effect of the transcription factor WT1 mediated by lncRNA WT1-AS on the oxidative stress injury and apoptosis of neurons in AD via *in vitro* and *in vivo* experiments.

## RESULTS

### LncRNA WT1-AS was poorly expressed in SH-SY5Y cells treated with Aβ_25-35_ and inhibited OSI and apoptosis induced by Aβ_25-35_

**Figure 1:**
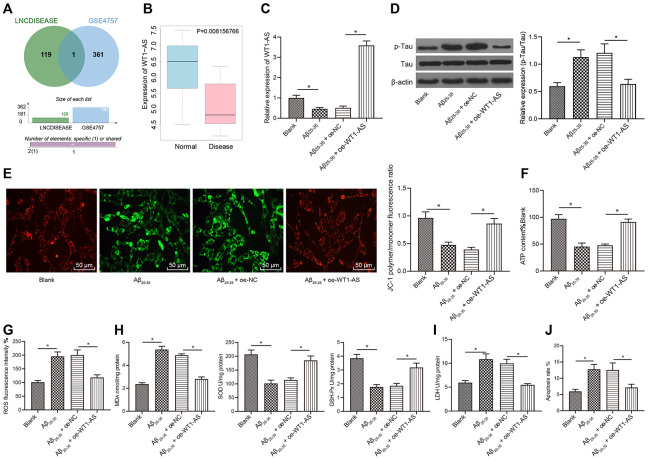
**WT1-AS inhibited OSI and apoptosis of SH-SY5Y cells treated with Aβ_25-35_** (**A**) The two circles in the figure represent the differential expression of lncRNAs in GSE4757 and lncRNAs related to AD obtained from the LINCDISEASE database, respectively, and the middle part represents the intersection of the two datasets. (**B**) WT1-AS expression in GSE4757; abscissa, sample type; ordinate, gene expression; blue box, normal sample; and red box, tumor sample. (**C**) The expression of WT1-AS measured by qRT-PCR. (**D**) The expression of p-Tau and total Tau detected by western blot. (**E**) Detection of mitochondrial membrane potential by JC-1 staining (200x). (**F**) Detection of ATP content. (**G**) Detection of ROS content. (**H**) Detection of MDA content, SOD and GSH-Px activities. (**I**) Detection of LDH activity. (**J**) Detection of apoptosis by flow cytometry. *P<0.05; the experimental results are expressed as the mean ± standard deviation. Differences among multiple groups were analyzed using one-way ANOVA followed by Tukey’s post hoc test. The experiment was repeated three times.

Initial analysis of the LINCDISEASE database identified a total of 120 lncRNAs implicated in AD. In addition, differential gene expression analysis of the AD-related GSE4757 dataset revealed 362 genes with significantly different expression. Subsequent Venn diagram analysis of the 362 differentially expressed genes with the prediction results of the LINCDISEASE database (Figure 1A) identified one lncRNA at the intersection between the datasets, namely, WT1-AS. The expression profile of WT1-AS in the GSE4757 dataset was analyzed, which demonstrated that WT1-AS had markedly reduced expression in AD (Figure 1B). Additionally, SH-SY5Y cells treated with Aβ_25-35_ were employed to construct an *in vitro* AD cell model. The qRT-PCR results also revealed that the expression of WT1-AS was significantly decreased following Aβ_25-35_ treatment; however, the expression of WT1-AS in SH-SY5Y cells treated with Aβ_25-35_ was markedly elevated after overexpressing WT1-AS (Figure 1C). Moreover, Aβ_25-35_ treatment significantly promoted Tau protein phosphorylation, but without an obvious effect on the protein expression of total Tau (Figure 1D), decreased the mitochondrial membrane potential (Figure 1E), and inhibited ATP production (Figure 1F), while WT1-AS overexpression reversed these effects of Aβ_25-35_. Furthermore, after Aβ_25-35_ treatment, the levels of ROS (Figure 1G), MDA (Figure 1H) and LDH activity (Figure 1I) in SH-SY5Y cells were found to significantly increase, while the levels of SOD and GSH-Px were clearly reduced; the apoptosis of SH-SY5Y cells increased significantly after Aβ_25-35_ treatment, as evidenced by flow cytometry assay results (Figure 1J). However, overexpression of WT1-AS in SH-SY5Y cells treated with Aβ_25-35_ reversed the effect of Aβ_25-35_.

### LncRNA WT1-AS inhibited OSI and apoptosis by decreasing the expression of WT1

Existing studies have highlighted the downstream regulatory mechanism of WT1-AS, wherein WT1-AS inhibits the expression of the WT1 gene [37], and these findings were further verified in SH-SY5Y cells.

**Figure 2:**
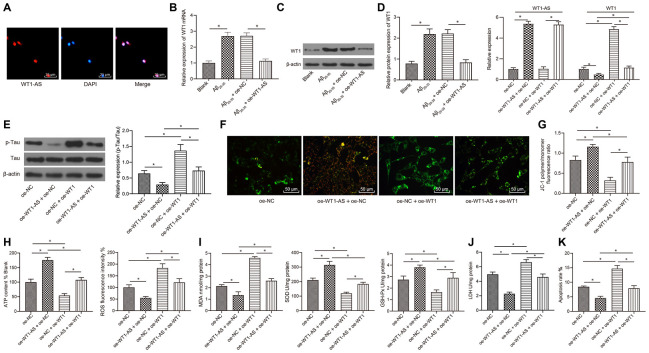
**LncRNA WT1-AS inhibited OSI and apoptosis by inhibiting the expression of WT1.** (**A**) Subcellular localization of WT1-AS in SH-SY5Y cells determined by FISH (400x). (**B**) The expression of WT1 measured by qRT-PCR. (**C**) The expression of WT1 detected by WB. (**D**) The expression of WT1-AS and WT1 measured by qRT-PCR. (**E**) The expression of p-Tau and total Tau detected by western blot. (**F**) Detection of mitochondrial membrane potential by JC-1 staining (200x). (**G**) Detection of ATP content. (**H**) Detection of ROS content. (**I**) Detection of MDA content, SOD and GSH-Px activities. (**J**) Detection of LDH activity. (**K**) Detection of apoptosis by flow cytometry. *P<0.05; the experimental results are expressed as the mean ± standard deviation. Differences among multiple groups were analyzed using one-way ANOVA followed by Tukey’s post hoc test. The experiment was repeated three times.

Subcellular localization of WT1-AS was detected using FISH assay, and WT1-AS was found to be primarily expressed in the nucleus (Figure 2A). After overexpressing WT1-AS in Aβ_25-35_-treated SH-SY5Y cells, qRT-PCR and WB detection results showed that the expression of WT1 increased significantly after Aβ_25-35_ treatment but decreased noticeably after WT1-AS overexpression (Figure 2B, 2C).

Previous studies have also shown that WT1 is highly expressed in AD and can further promote apoptosis and lead to neurological failure [38]. As a result, we speculated that overexpression of WT1-AS could inhibit the development of AD by suppressing WT1, which was verified by further experimentation. In the current study, WT1-AS and WT1 were overexpressed in SH-SY5Y cells treated with Aβ_25-35_ (Figure 2D). Furthermore, compared with that in the oe-NC group, the expression of WT1-AS was greatly increased, while the expression of WT1 was obviously decreased in the oe-WT1-AS+oe-NC group. There were no significant differences in the expression of WT1-AS between the oe-NC+oe-WT1 group and oe-NC group, whereas significantly elevated expression of WT1 was observed in the oe-NC+oe-WT1 group. However, overexpression of WT1 reversed the effect of WT1-AS overexpression on WT1. Moreover, overexpression of WT1-AS in Aβ_25-35_-treated SH-SY5Y cells could significantly inhibit Tau protein phosphorylation, without obvious effects on the protein expression of total Tau (Figure 2E), increase mitochondrial membrane potential (Figure 2F), and promote ATP production (Figure 2G), while overexpression of WT1 reversed the above effect of WT1-AS overexpression. Further detection of OSI in SH-SY5Y cells demonstrated that after overexpressing WT1-AS, the levels of ROS (Figure 2H) and MDA (Figure 2I) as well as LDH activity (Figure 2J) in SH-SY5Y cells decreased significantly, while the levels of SOD and GSH-Px were markedly elevated. Moreover, flow cytometry results showed that the apoptosis of SH-SY5Y cells decreased significantly after overexpressing WT1-AS (Figure 2K), while overexpression of WT1 reversed the above effect of WT1-AS overexpression.

### LncRNA WT1-AS inhibited miR-375 expression by regulating transcription factor WT1

**Figure 3:**
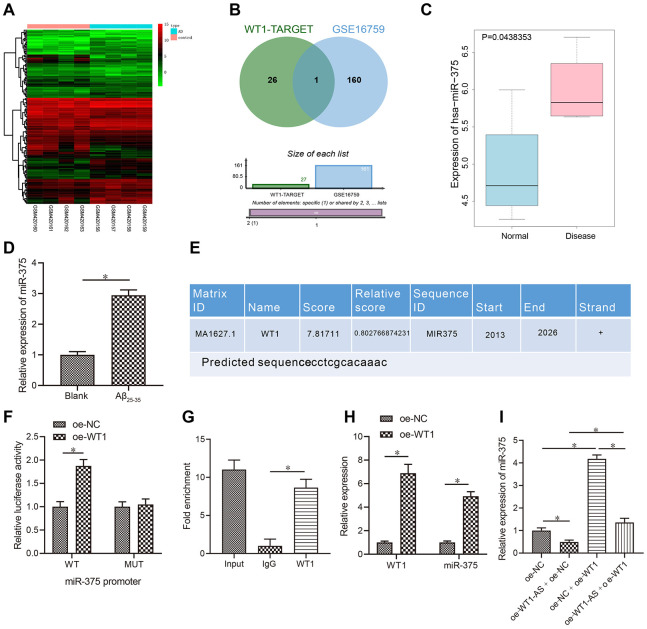
**LncRNA WT1-AS inhibited miR-375 expression by regulating the transcription factor WT1.** (**A**) Differential miRNA expression heatmap of GSE16759; abscissa, sample number; ordinate, miRNA name; and left tree, miRNA expression level clustering. Each small square in the figure represents the expression of one miRNA in a sample, and the histogram at the top right is the color scale. (**B**) Prediction of regulatory miRNAs downstream of WT1. The middle part indicates the intersection of predicted WT1 target miRNAs and differentially expressed miRNAs in GSE16759. (**C**) The differential expression of miR-375 in GSE16759. (**D**) The expression of miR-375 in Aβ_25-35_treated SH-SY5Y cells detected by qRT-PCR. (**E**) Prediction of WT1 binding site in the miR-375 promoter region by combining NCBI (https://www.ncbi.nlm.nih.gov/) and JASPAR (http://jaspar.genereg.net/). (**F**) After mutation of WT1 in the promoter region of miR-375, dual luciferase reporter assay was used to detect WT1 targeted binding to the promoter region of miR-375 in HEK-293T cells. (**G**) Detection of WT1 targeted binding to the promoter region of miR-375 by ChIP assay. (**H**–**I**) The expression of miR-375 detected by qRT-PCR. *P<0.05; the experimental results are expressed as the mean ± standard deviation. Differences among multiple groups were analyzed using one-way ANOVA followed by Tukey’s post hoc test. The experiment was repeated three times.

According to the prediction results of the WT1 downstream mechanism, WT1 could increase or decrease the expression of miRNAs to a significant degree. In the study carried out by Su et al. [39], 27 potential regulatory miRNAs were predicted to function downstream of WT1. One miRNA expression dataset, GSE16759, was retrieved from the GEO database, and the analysis results yielded a total of 161 miRNAs with significant differential expression (Figure 3A). Venn diagram analysis of these 161 miRNAs with significantly different expression levels and the aforementioned prediction results was performed (Figure 3B), which revealed just one common miRNA, miR-375, that was also highly expressed in AD (Figure 3C). The qRT-PCR results further revealed that miR-375 was highly upregulated in Aβ_25-35_-treated cells (Figure 3D). Furthermore, the binding site of WT1 in the promoter region of miR-375 was predicted using NCBI and Jaspar databases (http://jaspar.genereg.net/) (Figure 3E). Next, the binding site was mutated, and while overexpression of WT1 significantly increased the luciferase activity of the wild-type promoter of miR-375 in HEK-293T cells, it had no effects on the mutant, indicating that WT1 binds directly to the gene promoter of miR-375 (Figure 3F). In addition, ChIP assay results also indicated that WT1 binds directly to the promoter region of miR-375 (Figure 3G). Following overexpression of WT1 in SH-SY5Y cells treated with Aβ_25-35_, miR-375 was found to be overexpressed after WT1 overexpression (Figure 3H). However, following simultaneous overexpression of WT1-AS and WT1 in SH-SY5Y cells treated with Aβ_25-35_, the expression of miR-375 decreased significantly after WT1-AS overexpression, while overexpression of WT1-AS and WT1 reversed the effect of WT1-AS overexpression alone (Figure 3I).

### LncRNA WT1-AS/WT1 inhibited OSI and apoptosis by inhibiting miR-375 expression

**Figure 4:**
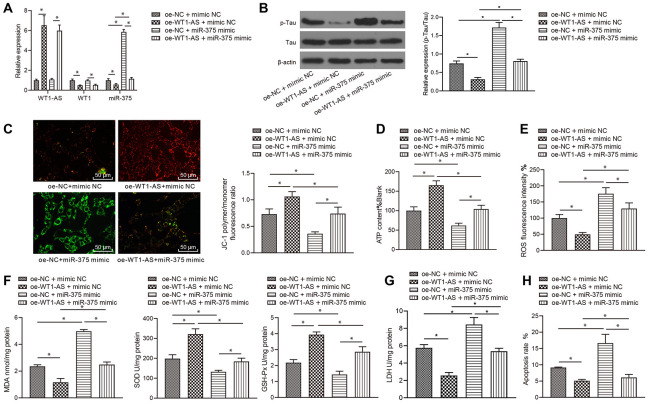
**LncRNA WT1-AS/WT1 inhibited OSI and apoptosis by inhibiting miR-375 expression.** (**A**) The expression of WT1-AS, WT1 and miR-375 detected by qRT-PCR. (**B**) The expression of p-Tau and total Tau detected by western blot. (**C**) Detection of mitochondrial membrane potential by JC-1 staining. (**D**) Detection of ATP content. (**E**) Detection of ROS content. (**F**) Detection of MDA content, SOD and GSH-Px activities. (**G**) Detection of LDH activity. (**H**) Detection of apoptosis by flow cytometry. *P<0.05; the experimental results are expressed as the mean ± standard deviation. Differences among multiple groups were analyzed using one-way ANOVA followed by Tukey’s post hoc test. The experiment was repeated three times.

After showing that WT1-AS could inhibit the expression of miR-375 by regulating WT1, we speculated that the regulation by WT1-AS/WT1 of the development of AD was mediated by miR-375. To verify our hypothesis, WT1-AS and miR-375 were overexpressed in SH-SY5Y cells treated with Aβ_25-35_, and the expression of WT1-AS, WT1 and miR-375 was subsequently detected by qRT-PCR. Overexpression of WT1-AS significantly increased the expression of WT1-AS and obviously decreased the expression of WT1 and miR-375, while simultaneous overexpression of WT1-AS and miR-375 reversed the effect of WT1-AS overexpression alone (Figure 4A). Moreover, overexpression of WT1-AS in Aβ_25-35_-treated SH-SY5Y cells significantly inhibited Tau protein phosphorylation but without a significant effect on the protein expression of total Tau (Figure 4B), increased mitochondrial membrane potential (Figure 4C), and promoted ATP production (Figure 4D), while simultaneous overexpression of WT1-AS and miR-375 reversed the effect of WT1-AS overexpression alone. Further detection of OSI in SH-SY5Y cells showed that after overexpressing WT1-AS, the levels of ROS (Figure 4E) and MDA (Figure 4F) as well as LDH activity (Figure 4G) in SH-SY5Y cells decreased significantly, while the levels of SOD and GSH-Px (Figure 4G) were markedly elevated. Moreover, the flow cytometry results demonstrated that the apoptosis of SH-SY5Y cells decreased significantly after WT1-AS overexpression alone (Figure 4H), while simultaneous overexpression of WT1-AS and miR-375 reversed this effect on apoptosis.

### miR-375 promoted OSI and apoptosis by inhibiting the expression of SIX4

**Figure 5:**
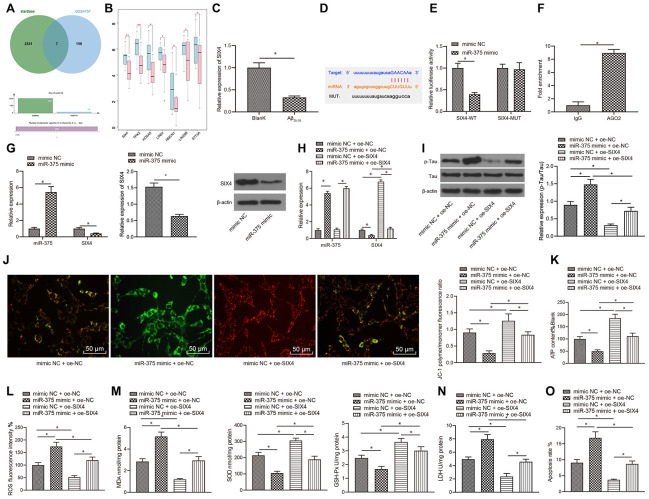
**miR-375 promoted OSI and apoptosis by inhibiting the expression of SIX4.** (**A**) Prediction of the target genes of miR-375, with the middle part representing the intersection of target prediction results and downregulated genes in GSE4757. (**B**) Differential expression of candidate target genes in GSE4757 (*p<0.05, **p<0.01). (**C**) The expression of SIX4 detected by qRT-PCR in Aβ_25-35_-treated SH-SY5Y cells. (**D**) Bioinformatic analysis predicted the presence of a binding site between miR-375 and SIX4 mRNA. (**E**) Dual luciferase reporter assay showed that miR-375 directly bound SIX4 mRNA. (**F**) RIP assay proved that miR-375 directly bound to SIX4. (**G**) The mRNA and protein expression of SIX4 after overexpression of miR-375 detected by qRT-PCR and western blot. (**H**) The expression of miR-375 and SIX4 detected by qRT-PCR. (**I**) The expression of p-Tau and total Tau detected by western blot. (**J**) Detection of mitochondrial membrane potential by JC-1 staining. (**K**) Detection of ATP content. (**L**) Detection of ROS content. (**M**) Detection of MDA content, SOD activity and GSH-Px activity. (**N**) Detection of LDH activity. (**O**) Detection of apoptosis by flow cytometry. *P<0.05; the experimental results are expressed as the mean ± standard deviation. Differences among multiple groups were analyzed using one-way ANOVA followed by Tukey’s post hoc test. The experiment was repeated three times.

To further elucidate the downstream regulatory mechanism of miR-375, the downstream target genes of miR-375 were predicted through the starBase database, which revealed a total of 2,338 potential regulatory target genes. The 2,338 target genes intersected with the significantly downregulated genes in GSE4757, and 7 candidate genes were finally obtained (Figure 5A). The 7 candidate genes in GSE4757 were all significantly downregulated in AD (Figure 5B), wherein the differential expression of the SIX4 and TPX2 genes was the most significant in normal samples and disease samples (differential p values of 0.0055 and 0.007, respectively); thus, SIX4 was selected as the research object. The expression pattern of SIX4 in SH-SY5Y cells treated with Aβ_25-35_ was detected with qRT-PCR, and SIX4 was found to be poorly expressed in cells treated with Aβ_25-35_ (Figure 5C). Additionally, bioinformatic analysis predicted the presence of binding sites between miR-375 and SIX4 mRNA (Figure 5D). Moreover, dual luciferase reporter assay and RIP assay results confirmed that miR-375 and SIX4 mRNA could indeed bind together (Figure 5E, 5F). In addition, both qRT-PCR and western blot assay results indicated that overexpression of miR-375 significantly inhibited the mRNA and protein expression of SIX4 (Figure 5G).

Subsequently, miR-375 and SIX4 were overexpressed in SH-SY5Y cells treated with Aβ_25-35_ (Figure 5H). According to the results, overexpression of miR-375 in SH-SY5Y cells treated with Aβ_25-35_ significantly promoted Tau protein phosphorylation, without an obvious effect on the protein expression of Tau (Figure 5I), reduced mitochondrial membrane potential (Figure 5J), and inhibited ATP production (Figure 5K), while overexpression of miR-375 and SIX4 reversed the effects of miR-375 overexpression alone. Further detection of OSI in SH-SY5Y cells demonstrated that the levels of ROS (Figure 5 L) and MDA (Figure 5 M) as well as LDH activity (Figure 5N) in SH-SY5Y cells were all remarkably increased, while SOD and GSH-Px levels (Figure 5N) were obviously decreased after miR-375 overexpression. In addition, apoptosis was found to be significantly increased following miR-375 overexpression (Figure 5O), whereas simultaneous overexpression of miR-375 and SIX4 reversed the effect of single miR-375 overexpression.

### WT1-AS inhibited the miR-375/SIX4 axis to suppress OSI and apoptosis by regulating the transcription factor WT1

**Figure 6:**
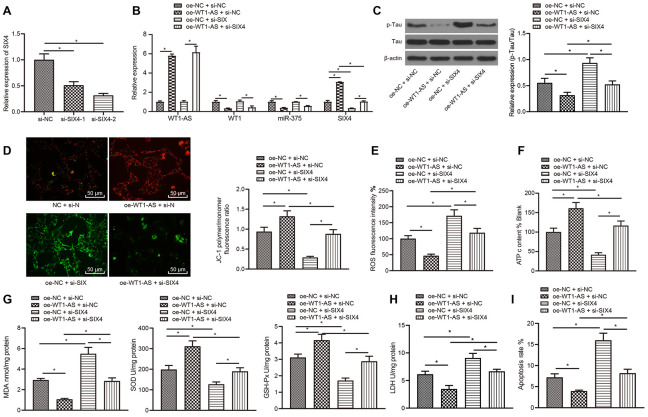
**WT1-AS can inhibit the miR-375/SIX4 axis to suppress OSI and apoptosis by downregulating the transcription factor WT1.** (**A**) The expression of SIX4 detected by qRT-PCR in cells transfected with SIX4-1 and SIX4-2 to screen a siRNA sequence. (**B**) The expression of WT1-AS, WT1, miR-375 and SIX4 detected by qRT-PCR. (**C**) The expression of p-Tau and total Tau detected by western blot. (**D**) Detection of mitochondrial membrane potential by JC-1 staining. (**E**) Detection of ATP content. (**F**) Detection of ROS content. (**G**) Detection of MDA content, SOD and GSH-Px activities. (**H**) Detection of LDH activity. (**I**) Detection of apoptosis by flow cytometry. *P<0.05; the experimental results are expressed as the mean ± standard deviation. Differences among multiple groups were analyzed using one-way ANOVA followed by Tukey’s post hoc test. The experiment was repeated three times.

The abovementioned results indicated that lncRNA WT1-AS inhibited the expression of miR-375 by suppressing WT1 to suppress the occurrence and development of AD. In addition, miR-375 could also promote the occurrence and development of AD by inhibiting the expression of SIX4. Thus, we investigated whether lncRNA WT1-AS could inhibit the development of AD through WT1 inhibition. According to the results in subsequent experiments, WT1-AS was overexpressed and SIX4 was silenced in SH-SY5Y cells treated with Aβ_25-35_ (Figure 6A, 6B). It was found that overexpression of WT1-AS in Aβ_25-35_-treated SH-SY5Y cells significantly inhibited Tau protein phosphorylation without a significant effect on the protein expression of total Tau (Figure 6C), increased mitochondrial membrane potential (Figure 6D), and promoted ATP production (Figure 6E), while SIX4 silencing reversed the effects of WT1-AS overexpression. Further detection of OSI in SH-SY5Y cells showed that after overexpression of WT1-AS, the levels of ROS (Figure 6F) and MDA (Figure 6G) as well as LDH activity (Figure 6H) were all significantly decreased in SH-SY5Y cells, while the levels of SOD and GSH-Px were markedly increased. Moreover, the results of flow cytometry showed that the apoptosis of SH-SY5Y cells decreased significantly after overexpression of WT1-AS (Figure 6I), whereas SIX4 silencing reversed the effects of WT1-AS overexpression.

### WT1-AS inhibited OSI and apoptosis of neurons in AD *in vivo*

**Figure 7:**
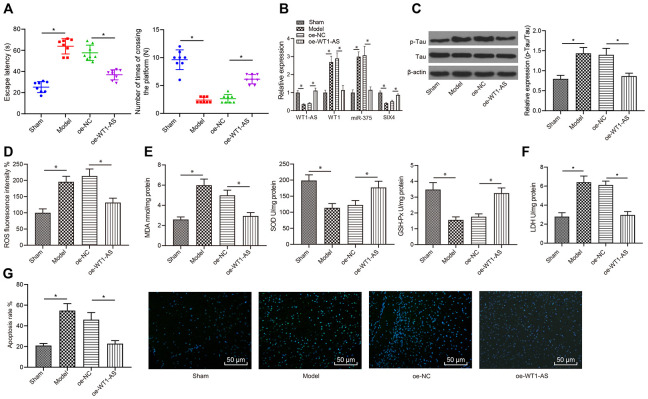
**WT1-AS inhibited OSI and apoptosis of neurons in AD *in vivo*.** (**A**) Detection of the learning and memory abilities of mice by using the Morris water maze test. (**B**) The expression of WT1-AS, WT1, miR-375 and SIX4 detected by qRT-PCR in brain tissues of mice. (**C**) The expression of p-Tau and total Tau detected by western blot. (**D**) Detection of ROS content. (**E**) Detection of MDA content, SOD and GSH-Px activities. (**F**) Detection of LDH activity. (**G**) Detection of apoptosis by TUNEL staining (400x). *P<0.05; the experimental results were expressed as the mean ± standard deviation. Differences among multiple groups were analyzed using one-way ANOVA followed by Tukey’s post hoc test, n = 10 mice.

Finally, *in vivo* experiments were carried out for further validation of the abovementioned results. AD mouse models were established to test the learning and memory abilities of mice in each group with the Morris water maze test. Compared with those of the sham group, the learning and memory abilities of the model group were markedly decreased, and overexpression of WT1-AS improved the learning and memory abilities of AD mice (Figure 7A). Following the collection of mouse brain tissues, qRT-PCR analysis revealed that the expression levels of WT1-AS and SIX4 were obviously lower in AD mice, while those of WT1 and miR-375 were significantly elevated. In contrast, overexpression of WT1-AS significantly promoted the expression of SIX4 but inhibited the expression of WT1 and miR-375 (Figure 7B). At the same time, Tau protein phosphorylation levels were significantly increased in AD mice, with no remarkable effect on the protein expression of Tau (Figure 7C), accompanied by clearly increased levels of ROS (Figure 7D) and MDA (Figure 7E) as well as LDH activity (Figure 7F), but obviously lower levels of SOD and GSH-Px (Figure 7F). Following WT1-AS overexpression, there were significant differences, which manifested as decreased Tau protein phosphorylation levels, increased ROS and MDA levels, and decreased activities of SOD, GSH-Px and LDH. Moreover, TUNEL fluorescent staining results demonstrated that apoptosis in the hippocampus of AD mice was significantly increased (Figure 7G), which could be reduced by WT1-AS overexpression.

## DISCUSSION

Early and timely detection and diagnosis are the cornerstones to improving the prognosis of human malignant diseases [40], which highlights the need for novel detection methods that provide more flexibility and accuracy than traditional methods. Comprehensive treatment modalities such as radiotherapy, chemotherapy and surgery have been the mainstay of treatment for human malignancies for a long time [41, 42]. However, with advancements in molecular biotechnology, the medical field has seen the advent of novel molecular targeted therapies over the last decade [43]. For instance, abnormal expression of multiple miRNAs has been revealed in cancer cells, and miRNA regulation could prove to be highly beneficial for treating malignancies [44]. MicroRNAs (miRNAs) possess the ability to upregulate or downregulate the posttranscriptional expression of numerous genes by binding to the complementary 3′-untranslated regions of their target genes and playing critical roles in several biological and metabolic processes in various species [45, 46]. A great number of studies have also investigated the relationship of miRNAs in the progression of AD. One such study by Geekiyanage H et al. discovered a loss of miR-137, miR-181c, miR-9, and miR-29a/b-1 in AD, which could increase SPT and in turn Aβ levels, which represent intrinsic mechanisms for elevated risk of AD, suggesting the use of miRNAs as potential therapeutic targets for sporadic AD [47]. Wang X et al. also reported the involvement of miR-34a in AD pathogenesis, which was partially attributed to Bcl2 upregulation [48]. In addition, a recent study by Jin Y revealed that by augmenting the expression of inflammatory factors and promoting oxidative stress through suppression of SphK1, miR-125b may promote the development of AD and stimulate neuronal cell growth and apoptosis [49]. Another key molecule of interest, lncRNAs, are also abnormally expressed in various types of diseases [28]. These lncRNAs primarily exist in the nucleus, participate in the basic process of gene regulation, including chromatin, modification and direct transcription regulation, and regulate posttranscriptional events (splicing, editing localization, translation and degradation) [50, 51]. Multiple previous experiments also support and validate the participation of lncRNAs in the development of AD. For instance, a recent study carried out by Zhou M revealed age- and disease-dependent region-specific lncRNA expression patterns in aging and AD, further citing the use of LncSigAD9 identification to differentiate between AD and healthy controls with high diagnostic sensitivity and specificity. These scientific gains highlight the importance of lncRNAs in brain aging and AD, which can serve as promising biomarkers for the diagnosis and treatment of AD at earlier stages [52]. Similarly, the identification and qualitative research on miRNAs and lncRNAs has broadened the view of the critical roles of miRNAs and lncRNAs in the proliferation, differentiation, apoptosis and cell cycle regulation of malignant tumors [53, 54]. One such miRNA, miR-375, originally considered a pancreatic-specific miRNA [55, 56], has been found to be abnormally expressed in various human cancers. Moreover, the WT1-AS group of lncRNAs has also been shown to exert its effects on gastric cancer with both tumor suppressor and oncogenic activities [57]. Nevertheless, there are still a limited number of studies focusing on the role, mechanism and relationship of miR-375 and WT1-AS in the development of AD, which highlights the significance and innovation of our study in one aspect, but future research is certainly also needed to verify these findings from multiple perspectives.

First, we constructed an AD model by using Aβ25-35, which has been commonly used for AD modeling [58, 59]. Aβ1-42 mostly exists in soluble monomers, and while it is more toxic and prone to aggregation in AD, it does not have neurotoxicity; however, neurotoxicity develops usually when the equilibrium is broken and Aβ monomers gradually form oligomers or condensed structures. Aβ25-35 is a fragment obtained from the hydrolysis of the Aβ protein *in vitro* that does not exist *in vivo*. However, its neurotoxicity is almost equal to that of the full-length fragment of endogenous Aβ, and it has been confirmed that the key site of Aβ polymerization and its toxic fragment are located in the middle part of Aβ. Initial findings from microarray analysis and database prediction indicated that low expression of lncRNA WT1-AS in AD was likely to participate in the regulation of AD. Further mechanistic prediction revealed that lncRNA WT1-AS could further inhibit the miR-375/SIX4 axis through WT1 to influence the fate of AD. Subsequent *in vitro* experiments in the current study demonstrated that lncRNA WT1-AS was poorly expressed in Aβ_25-35_-treated SH-SY5Y cells, whereas overexpression of WT1-AS resulted in significant inhibition of WT1 expression. Dual luciferase reporter assay and ChIP experimental findings also illustrated that WT1 could target the promoter region of miR-375 to promote its expression, wherein miR-375 could bind with the SIX4 mRNA, and overexpressing miR-375 significantly inhibited the expression of SIX4, confirming that WT1-AS can inhibit miR-375 expression by regulating WT1.

Mitochondria are not only intracellular energy converters but also important factors determining cell survival and apoptosis. Mitochondrial damage and dysfunction are closely related to the occurrence and development of many neurodegenerative diseases. In this study, when WT1-AS was overexpressed, lncRNA WT1-AS inhibited WT1, WT1-AS/WT1 suppressed miR-375 expression, and inhibiting miR-375 promoted SIX4 expression, all of which could inhibit Tau protein phosphorylation and promote ATP production, thus participating in the regulation of mitochondrial structure and function. These observations may provide a new idea for studying the pathogenesis and potential treatment of AD. Furthermore, our findings demonstrated that WT1-AS could inhibit OSI and apoptosis induced by Aβ_25-35_, while overexpression of WT1 and miR-375 or SIX4 silencing reversed these effects of WT1-AS on OSI and apoptosis. Additionally, the results of the *in vivo* experiment showed that WT1-AS could improve the learning and memory abilities of AD model mice and inhibit OSI and apoptosis. WT1-AS is the antisense transcript of WT1, and our investigations demonstrated for the first time that WT1-AS/WT1 could suppress cell OSI and apoptosis by inhibiting the expression of miR-375, whereas miR-375 promoted OSI and apoptosis by inhibiting the expression of SIX4. More importantly, we revealed that WT1-AS can inhibit the expression of WT1 and then suppress the miR-375/SIX4 axis to inhibit cell OSI and apoptosis, which could have detrimental effects on AD outcomes. Finally, through *in vivo* experiments, we show that WT1-AS could indeed inhibit neuronal OSI and apoptosis in AD mice *in vivo*. Collectively, the aforementioned findings shed new light on the mechanism of WT1-AS in AD. More specifically, our research demonstrated that WT1-AS was poorly expressed in AD, and the low expression levels of WT1-AS promoted the expression of the transcription factor WT1, which in turn increased miR-375 and inhibited the expression of SIX4, thereby promoting OSI and apoptosis of neurons in AD.

The pathogenesis of AD is highly complex, and numerous theories have been proposed, such as central cholinergic injury, microtubule-associated protein Tau protein abnormality theory, Aβ cascade theory, gene mutation or polymorphism theory, immune function mutation, and excitatory amino acid toxicity theory [60, 61]. There are large quantities of Aβ deposits in the brain tissues of AD patients, and these deposits damage the cell membrane, synapses and axons and consequently participate in the pathogenesis of AD [62, 63]. Therefore, reducing the production of Aβ or promoting its degradation might hold the key to effective AD treatment regimens. Our study incorporated the inhibition of specific genes or their mRNAs complementary to lncRNA to prevent the neurotoxic effect linked to Aβ.

In summary, the current study revealed the low expression of WT1-AS in AD, which promotes WT1 expression, stimulates miR-375 expression and inhibits SIX4 expression, thus promoting OSI and apoptosis of neurons in AD. In addition, we demonstrated that overexpression of WT1-AS significantly inhibits the miR-375/SIX4 axis, OSI and apoptosis of neurons in AD by suppressing the expression of WT1 in both *in vitro* and *in vivo* experimental models. Taken together, our data suggest that upregulation or downregulation of WT1-AS can have positive or negative effects on the development of AD and warrants further investigation to reduce the devastating effect of AD on patients and medical facilities. Regardless, in view of the existing limitations of our study, such as the lack of relevant controls for Aβ1-42, we plan to use Aβ1-42 treatment in future experiments to further verify our conclusion when funds and other objective conditions allow it.

## MATERIALS AND METHODS

### Bioinformatic analysis

First, lncRNAs associated with AD were retrieved from the LINCDISEASE 2.0 database (http://www.rnanut.net/lncrnadisease/index.html). Subsequent screening with a score > 0.2 of the prediction results using the clear prediction method identified a total of 120 human lncRNAs. In addition, the expression dataset GSE4757 and miRNA expression dataset GSE16759 were obtained from the GEO database (https://www.ncbi.nlm.nih.gov/geo/). The GSE4757 dataset comprised 10 normal samples and 10 diseased samples, while GSE16759 included 4 normal samples and 4 diseased samples. Next, differential analysis was performed to screen the differentially expressed genes among the two aforementioned datasets with the help of the “limma” package of R language with |logFC| > 1 and *p* value < 0.05. Finally, the downstream regulatory genes and binding sites of miR-375 were obtained from the StarBase database (https://www.lncrnablog.com/tag/starbase-v2-0/).

### Construction of lentiviral expression vectors

Expression vectors of WT1-AS, WT1 and SIX4 were constructed using a lentivirus packaging system. The entire sequences of WT1-AS (NR_023920.1), WT1 (NM_000378.6), SIX4 (NM_017420.5) and mouse WT1-AS (NR_015462.1) were synthesized with the XbaI and BamHI restriction sites (designed by BLOCK-iT™ RNAi Designer). T4 ligase was used to ligate the sequences to the lentivirus expression vector pLV-Puro (Cat. No. VL3001, Chongqing Yingmao Shengye Biotechnology Co., Ltd., China) [57, 64]. Lentivirus particles with high titer replication defects were produced after the recombinant pLV-puro vector and the packaging vector were cotransfected with HEK293T cells (packaging system Cat. No. klv3501). In addition, synthetic and chemically modified short RNA oligonucleotides were purchased from Suzhou Ribo Life Science [65], and the sequences of si-SIX4-1, si-SIX4-2 and si-NC are shown in Table 1.

**Table 1:** Information on siRNA sequences.

**Gene name**	**Sequence**
si-SIX4-1	5’-GGAGCTCTACAAGCAGAAT-3’
si-SIX4-2	5’-TCAAGGAGAAGTCGCGCAA-3’
si-NC	5’-TTCTCCGAACGTGTCACGT-3’

### Cell culture and transfection

SH-SY5Y human neuroblastoma cells were cultured in 1:1 Eagle’s minimal essential medium (EMEM):Ham’s F-12 medium (Sigma-Aldrich) supplemented with 10% FBS (ATCC). SH-SY5Y cells were differentiated using a modified version of a previously published protocol [66]. Briefly, SH-SY5Y cells were plated on fibronectin-coated plates (BD Biosciences, San Jose, CA, USA) and treated with 10 μM retinoic acid (Sigma-Aldrich) in 1:1 EMEM:Ham’s F-12 medium supplemented with 10% FBS for 7 days. SH-SY5Y cells were then treated with 50 ng/mL brain-derived neurotrophic growth factor (Life Technologies) in 1:1 EMEM:Ham’s F-12 medium (without FBS) for 3 days [67].

Moreover, Aβ_25-35_ preparation and cell treatment [68] were carried out according to previous literature [69], with lyophilized Aβ_25-35_ (Sigma-Aldrich, St. Louis, USA) incubated at 37° C for 3 days to polymerize Aβ_25-35_. Subsequently, the polymerized Aβ_25-35_ was prepared at 100 mg/ml and stored at –20° C for further experimentation. Later, SH-SY5Y cells (20 μm) were treated with polymerized Aβ_25-35_ for 24 h to establish the AD cell model [68].

Next, the cells were divided into the following groups: the blank group (SH-SY5Y cells without Aβ_25-35_ treatment), Aβ_25-35_ group (SH-SY5Y cells with Aβ_25-35_ treatment), oe-NC group (Aβ_25-35_ treatment + oe-NC transfection), Aβ_25-35_+oe-WT1-AS group (Aβ_25-35_ treatment + oe-WT1-AS transfection), oe-WT1-AS+oe-NC group (Aβ_25-35_ treatment + cotransfection of oe-WT1-AS and oe-NC), oe-NC+oe-WT1 group (Aβ_25-35_ treatment + cotransfection of oe-WT1 and oe-NC), oe-WT1-AS+oe-WT1 group (Aβ_25-35_ treatment + cotransfection of oe-WT1-AS and oe-WT1), oe-WT1 (Aβ_25-35_ treatment + oe-WT1 transfection), oe-NC+mimic NC group (Aβ_25-35_ treatment + cotransfection of oe-NC and mimic NC), oe-WT1-AS+mimic NC group (Aβ_25-35_ treatment + cotransfection of oe-WT1-AS and mimic NC), oe-NC+miR-375 mimic group (Aβ_25-35_ treatment + cotransfection of oe-NC and miR-375 mimic), oe-WT1-AS+miR-375 mimic group (Aβ_25-35_ treatment + cotransfection of oe-WT1-AS and miR-375 mimic), mimic NC+oe-SIX4 group (Aβ_25-35_ treatment + cotransfection of oe-SIX4 and mimic NC), miR-375 mimic+oe-SIX4 group (Aβ_25-35_ treatment + cotransfection of miR-375 mimic and oe-SIX4), oe-NC+si-NC group (Aβ_25-35_ treatment + cotransfection of oe-NC and si-NC), oe-WT1-AS+si-NC group (Aβ_25-35_ treatment + cotransfection of oe-WT1-AS and si-NC), oe-NC+si-SIX4 group (Aβ_25-35_ treatment + cotransfection of oe-NC and si-SIX4), and oe-WT1-AS+si-SIX4 group (Aβ_25-35_ treatment + cotransfection of oe-WT1-AS and si-SIX4).

Transfection of si-SIX4-1, si-SIX4-2 and si-NC was carried out at a concentration of 100 nM. The transfection reactions were performed using RNAiMAX (Invitrogen) in Opti-MEM medium (prewarmed at 37° C) according to the manufacturer’s instructions. Moreover, oe-WT1-AS, oe-WT1, oe-SIX4 and other plasmids were transfected in a 24-well plate (concentration of 1 μg/well). Cell transfection of the plasmids was performed using the Lipofectamine 2000 reagent (Invitrogen) in Opti-MEM medium (prewarmed at 37° C) according to the manufacturer’s instructions [67]. Transfection of miR-375 mimic and mimic NC was also carried out at a concentration of 100 nM, with the corresponding transfection performed using the Lipofectamine 2000 reagent (Invitrogen) in Opti-MEM medium (prewarmed at 37° C) [65]. The sequence of the miR-375 mimic was F:5’-UUUGUUCGUUCGGCUCGCGUGA-3’, R:3’-AAACAAGCAAGCCGAGCGCACU-5’, and the sequence of mimic NC was F: 5’-UUUGUACUACACAAAAGUACUG-3’, R: 3’-AAACAUGAUGUGUUUUCAUGAC-5’ [70].

### qRT-PCR assay

Total RNA was extracted from cultured and transfected cells using RNeasy Mini kits (Qiagen, Valencia, CA, USA), which were used to obtain cDNA with the help of reverse transcription kits (RR047A, Takara, Japan). A miRNA First Strand cDNA Synthesis (Tailing Reaction) kit (B532451-0020, Sangon Biotech Co., Ltd., Shanghai, China) was also used to obtain cDNA for reverse transcription to detect miRNA expression profiles. Sampling was performed with SYBR Premix Ex Taq II (Perfect Real Time) kits (DRR081, Takara, Japan). qRT-PCR was conducted using a real-time qPCR instrument (ABI 7500, ABI, Foster City, CA, USA). The primers were synthesized by Sangon Biotech (the premier sequences were designed as shown in Table 2). CT values were recorded for each well, with U6 as the internal reference gene of miR-375 and GAPDH as the reference for the remaining indices. Finally, the relative expression levels were calculated using the 2^-ΔΔCt^ method. ΔΔCt = (mean CT value of the target gene in the experimental group - mean CT value of the housekeeping gene in the experimental group) - (mean CT value of the target gene in the control group - mean CT value of the housekeeping gene in the control group).

**Table 2:** Sequence information for qRT-PCR.

**Gene name**	**Sequence**
WT1-AS (human)	F: 5′-GCCTCTCTGTCCTCTTCTTTGT-3′
	R: 5′-GCTGTGAGTCCTGGTGCTTAG-3′
WT1 (human)	F: 5′-GCATCTGAGACCAGTGAGAAA-3′
	R: 5′-TCCTGCTGTGCATCTGTAAG-3′
SIX4 (human)	F: 5′-AGCAGCTCTGGTACAAGGC-3′
	R: 5′- CTTGAAACAATACACCGTCTCCT-3′
GAPDH (human)	F: 5′-TTGGCATCGTTGAGGGTCT-3′
	R: 5′-CAGTGGGAACACGGAAAGC-3′
miR-375 (human)	F: 5’-AGCCGTCAAGAGCAATAACGAA-3’
	R: 5’-GTGCAGGGTCCGAGGT-3’
U6 (human)	F: 5’-CTCGCTTCGGCAGCACA-3’
	R: 5’-AACGCTTCACGAATTTGCGT-3’
WT1-AS (mouse)	F: 5’-AGAAGGCAGGACAAGGAAAG-3’
	R: 5’-GGATTTCTCAAGCAGAGGGTAG-3’
WT1 (mouse)	F: 5’-GGAGCTACCTTAAAGGGAATGG-3’
	R: 5’-CGTGTGGTTCTCACTCTCATAC-3’
SIX4 (mouse)	F: 5’-CCTCCCAGGATGTGAAAGAA-3’
	R: 5’-CACTTCAGTGCAGGGTATCA-3’
GAPDH (mouse)	F: 5′-TGTGTCCGTCGTGGATCTGA-3′
	R: 5′-CCTGCTTCACCACCTTCTTGAT-3′
miR-375 (mouse)	F: 5′-AGCCGTTTGTTCGTT CGGCT-3′
	R: 5′-GTGCAGGGTCCGAGGT-3′
U6 (mouse)	F: 5′-CGCTTCGGCAGCACATATAC-3′
	R: 5′-TTCACGAATTTGCGTGTCAT-3′

### Western blot

Total protein was extracted from tissues and cells of each group, and the protein concentration was measured with BCA kits (Thermo, USA). Total proteins (30 μg) were separated by polyacrylamide gel electrophoresis at a constant voltage of 80 V for 35 min and then at 120 V for 45 min. After separation, proteins were transferred to a polyvinylidene difluoride membrane (Amersham, USA), which was blocked with 5% skimmed milk powder at room temperature for 1 h. After discarding the blocking solution, primary antibodies were added for incubation at 4° C overnight, which included rabbit anti-p-Tau (dilution ratio of 1:10000, ab109390, Abcam, UK), rabbit anti-WT1 (dilution ratio of 1:500, ab89901, Abcam, UK), and anti-β-actin (dilution ratio of 1:5000, ab8227, Abcam, UK). Subsequently, the membrane was rinsed three times (10 min each) with PBST buffer (PBS buffer containing 0.1% Tween-20). Then, goat anti-rabbit IgG antibody labeled with HRP was incubated at room temperature for 1 h, and the membrane was rinsed three times (10 min each) with PBST buffer. Following scanning and development with an optical luminescence instrument (GE, USA), the relative expression patterns of proteins were analyzed using Image-Pro Plus 6.0 software (Media Cybernetics, USA). The experiment was performed three times to obtain the mean values.

### JC-1 staining to detect mitochondrial membrane potential

Cells at the logarithmic growth phase were inoculated in 6-well culture plates with 2 ml culture medium per well, adjusted to a density of 6×10^4^ cells/ml, followed by two PBS rinses. Then, JC-1 at a final concentration of 1 μg/mL was added at 37° C to incubate cells for 0.5 h, followed by three PBS rinses. The fluorescence intensity of mitochondria was observed by fluorescence microscopy (ECLIPSE Ti, Nikon, Japan) using an excitation wavelength of 488 nm and emission wavelengths of 595 nm and 525 nm.

### ATP generation detection

Hippocampal tissues were extracted, weighed and quickly placed in cold normal saline, with the addition of approximately 100-200 μL lysis buffer to every 20 mg of tissues, and then homogenized with a glass homogenizer. After lysis, the supernatant was centrifuged at 4° C at 12,000 ×g for 5 min for subsequent ATP measurement. ATP assay procedures were carried out according to the instructions of the test kit (S0026, Beyotime).

### Determinations of ROS levels

ROS formation was detected with the help of 2',7'-dichlorodihydrofluorescein diacetate (DCFH-DA, Sigma chemicals Co., St. Louis, MO, USA). Nonfluorinated DCFH-DA was oxidized with hydroxyl, peroxyl and other ROS in cells to generate fluorescent DCF. Cells were then seeded in 96-well plates at a density of 2 × 10^4^ for microplate reading (Greiner Bio-one GmbH, Frickenhausen, Germany) and in chamber slides for fluorescence microscopy (Thermo Fisher, Waltham, MA, USA). After treatment with Aβ_25-35_, cells were incubated with 25 μM DCF-DA in medium at 37° C for 30 min in conditions away from light. ROS in cells represented by DCF was measured with an EVOS fluorescence microscope (ThermoFisher, Waltham, MA, USA) and an Infinite M200 microplate reader (Tecan, Männedorf, Switzerland). The excitation and emission wavelengths were 485 nm and 530 nm, respectively [71].

Additionally, 10 mg of mouse hippocampal tissues was obtained and resuspended in 1,000 μL PBS1X (containing 10 μL protease inhibitor; Amersham Life Science, Munich, Germany). The sample was subsequently tested for ROS according to the abovementioned steps.

### Determination of the activity of mitochondrial superoxide dismutase (SOD) and the levels of reduced glutathione (GSH) and malondialdehyde (MDA)

The hippocampus was extracted from the midbrain of mice of each experimental group, with 125 mm^3^ hippocampal tissues mixed with 1 ml PBS and centrifuged (12000 ×g) at 4° C for 10 min. After collecting the supernatant, the protein concentration was measured by BCA test kits (P0011, Beyotime), and the levels of MDA, SOD and GSH were tested with MDA (A003-1-2), SOD (A001-3-2), and GSH-Px (A005) kits, respectively, obtained from Nanjing Jiangcheng Bioengineering Institute (China).

SH-SY5Y cells were digested with trypsin, lysed at 4° C with an ultrasonic cell disruptor, and the lysate was centrifuged at 4° C for 10 min, with 100 μL of supernatant collected. The absorbance (OD) values were then detected with a microplate reader in accordance with the instructions of the SOD, GSH-Px, and MDA kits, and finally, the levels were calculated.

### Detection of lactate dehydrogenase (LDH) release rate

To evaluate the degree of cell damage, LDH activity was detected in the aforementioned supernatant using commercial kits. At the end of the different treatments, the tests were carried out in accordance with the manufacturer's instructions. The total OD values were measured at a wavelength of 490 nm. LDH test kits (A020-2-2) were obtained from the Nanjing Jiangcheng Bioengineering Institute (China).

### Flow cytometry

For apoptosis analyses, cells were rinsed with PBS and then treated with an Annexin V-FITC apoptosis detection kit (BD Biopharmingen, NJ, USA) in the dark for 15 min. All experiments were carried out by a BD Biosciences FACSCalibur Flow Cytometer (BD Biosciences, NJ, and USA). The experiment was repeated three times to obtain the mean value.

### FISH

Cells were briefly rinsed with PBS, fixed with 4% formaldehyde in PBS (pH 7.4) at room temperature for 15 min and then permeabilized with PBS containing 0.5% Triton X-100 on ice for 10 min. Prior to hybridization, cells were rinsed once with PBS and once with 2 × SSC. Anti-WT1-AS deoxynucleotide probes conjugated with Alexa Fluor 488 (Invitrogen, Carlsbad, CA) were hybridized for 16 h in a wet room at 50° C using a hybridization solution (probe dilution ratio of 1:1250) (Boster, China). For FISH, cells were washed at 50° C for 30 min with 25% deionized formamide/2 × SSC and then washed at 50° C for 30 min in 2 × SSC.

### Dual luciferase reporter assay

After restriction endonuclease digestion, T4 DNA ligase was used to insert the target fragment of the miR-375 gene promoter into the pGL3-basic reporter plasmid. Next, the WT and MUT luciferase reporter plasmids with correct sequences were cotransfected with oe-NC or oe-WT1 into HEK-293T cells (Shanghai Institute of Biochemistry, Chinese Academy of Sciences, Shanghai Research Center of Life Sciences, Shanghai, China). The SIX4 3'UTR gene fragment was artificially synthesized and introduced into the pMIR-REPORT vector (Promega, USA) using HindIII and BamHI endonucleases. The complementary mutation site of the seed sequence was introduced into the wild type SIX4 sequence. After restriction endonuclease digestion, T4 DNA ligase was used again to insert the target fragment into the pMIR-REPORT vector. After that, the WT and MUT luciferase reporter plasmids with correct sequences were cotransfected with miR-375 mimic into HEK-293T cells for 48 h. After cell lysis, the luciferase activity was detected using a Luminometer TD-20/20 detector (model: E5311, Promega, USA) according to the instructions of the Dual-Luciferase Reporter Assay System kit (Promega, USA). The experiment was repeated three times to obtain the mean value.

### ChIP assay

First, cells were fixed with formaldehyde for 10 min to induce DNA protein cross-linking. An ultrasonic processor was used to break the chromatin into fragments at time intervals of 10 s for 15 cycles. After that, mouse IgG (ab172730, dilution ratio of 1:1000, Abcam, Shanghai, China) and target protein-specific antibody anti-WT1 (sc-7385, 2μL per 500 μg of extract, Santa Cruz Biotechnology) were incubated at 4° C overnight for complete integration. The DNA protein complex was then precipitated with Protein Agarose/Sepharose, followed by centrifugation at 12,000 ×g for 5 min. After the supernatant was discarded, the nonspecific complex was washed, de-crosslinked overnight at 65° C, and DNA fragments were extracted and purified by phenol/chloroform. Next, the binding of WT1 and the miR-375 gene promoter was detected by qRT-PCR with miR-375 gene promoter-specific primers (F: 5’-GAAGACCAGGACCAGGAGAT-3’, R: 5’- GCTCAGGTCCGGTTTGTG -3’).

### RIP

The binding of miR-375 and SIX4 with AGO2 was detected using RIP kits (Millipore, USA), and the supernatant was discarded after rinsing with precooled PBS. Next, cells were lysed in an ice bath with equal volumes of RIP lysate for 5 min and centrifuged at 14,000 rpm for 10 min at 4° C. A portion of the cell extract was removed as input, and the remainder was incubated with antibody for coprecipitation, with the specific steps described as follows. First, 50 μL magnetic beads from each coprecipitation reaction system were suspended in 100 μL RIP Wash Buffer after cleaning. Then, 5 μg antibody was added and incubated to precipitate the bound complex according to the experimental grouping. After cleaning, the bead-antibody complex was resuspended in 900 μL RIP Wash Buffer and incubated overnight at 4° C with 100 μL cell extract. The following day, the sample was placed on the magnetic base to collect the bead-protein complex. The sample and the input were digested with protease K, and then the RNA was extracted for subsequent PCR detection. The antibody used in RIP was anti-AGO2 (ab32381, dilution ratio of 1:50, Abcam, UK), which was mixed at room temperature for 30 min, while IgG (ab109489, dilution ratio of 1:100, Abcam, UK) was used as the negative control. The relative levels of miR-375 and SIX4 were detected by qRT-PCR.

### AD modeling

A total of 40 BALB/c pure male mice (aged 6 weeks, weighing 16-18 g) were purchased from Shanghai SLAC Laboratory Animal Co., Ltd. and raised under SPF conditions. All experimental mice were kept in an experimental environment with constant temperature and humidity under an artificial light-dark cycle of 12 h of light (lights on at 08:00 and lights off at 20:00) with free access to feed and water. Then, the mice were divided into the sham group, model group (AD model), oe-NC group (AD model after oe-NC lentivirus injection), and oe-WT1-AS group (AD model after oe-WT1-AS lentivirus injection), with 10 mice in each group.

The establishment of AD mouse models was performed as previously described [72]. First, mice were intraperitoneally anesthetized with pentobarbital sodium (40 mg/kg) [73], followed by routine preparation of skin. After disinfection, the mice were fixed on the brain localizer to keep the anterior and posterior fontanelle at the same level, and then the fur in the middle of the mouse heads was removed to expose the anterior fontanelle. According to the Mouse Brain Atlas, with the Bregma point serving as the origin, a 5 ml syringe needle was inserted to pierce a small hole 3.0 mm behind the anterior fontanelle, 2.2 mm beside the midline, and 2.8 mm under the dura mater, followed by the insertion of a 10μL microinjector vertically and slow injection of 10 μL Aβ_25-35_ (2 mg/ml) for 10 min, with the needle retained for 10 min, to inject Aβ_25-35_ into the right ventricle. Following suturing of the scalp, the mice were injected with 4×10^4^ U penicillin every day for 3 days to prevent infection. In the sham operation group, 10 μL 0.9% sodium chloride solution was similarly injected into the lateral ventricles of mice, and the other treatment protocols were the same as those in the modeling groups.

In this process, 24 h prior to modeling, lentivirus (3 μl with similar titers > 1 × 10^9^ IU/ml) was delivered at a rate of 0.3 μl/min for stereotactic injection into the CA1 area of the hippocampus. Subsequent experimentation was performed 3 weeks after injection [74, 75]. All animal experiments were approved and supervised by the animal research committee of our institute and conformed to the animal experiment guidelines of our institute.

### Morris water maze test

Morris water maze testing was performed to assess the learning and memory abilities of mice. The maze test was divided into the following two parts: memory training test and memory retention test. For the memory training test, the midpoint of a quadrant was randomly selected as the starting point to test the time (escape incubation period) taken to find the platform within 60 s each time for 5 days. Each mouse was tested three times a day at intervals of at least 75 min. In the memory retention test, the number of times each mouse crossed the platform within 60 s was counted every other day after the last memory training test. Data acquisition and processing were completed using the Morris water maze automatic monitoring and processing system. The experiment was repeated three times to obtain the mean value.

### TUNEL staining

Hippocampal slices were rinsed twice with PBS, incubated with 0.3% H_2_O_2_ for 15 min, rinsed again with PBS for 15 min, and then incubated with TdT-mediated dUTP-biotin nick end-labeling (TUNEL) staining solution (C1086, Beyotime, Beijing, China) according to the manufacturer's instructions. The number of TUNEL-labeled positive cells was measured by means of laser scanning confocal microscopy (CS SP2; Leica, Wetzlar, Germany).

### Statistical analysis

All data were processed using SPSS 21.0 statistical software (SPSS, Inc., Chicago, IL, USA). Measurement data are expressed as the mean ± standard deviation. The paired-design data conforming to a normal distribution and homogeneity of variance between two groups were analyzed using a paired t-test. Data comparisons among multiple groups were performed using one-way analysis of variance (ANOVA), followed by Tukey’s post hoc test. A value of *p*<0.05 indicated statistical significance.

## Supplementary Material

Supplementary Figure 1
