# Supplementary material for: Effect of lncRNA WT1-AS regulating WT1 on oxidative stress injury and apoptosis of neurons in Alzheimer's disease via inhibition of the miR-375/SIX4 axis
Source: Aging (Albany NY). 2020 Nov 21;12(23):23974–95. doi: 10.18632/aging.104079 (PMC7762490; doi:10.18632/aging.104079)
Supplement: Supplementary Figure 1 [file aging-12-104079-s001.pdf]

SUPPLEMENTARY FIGURE

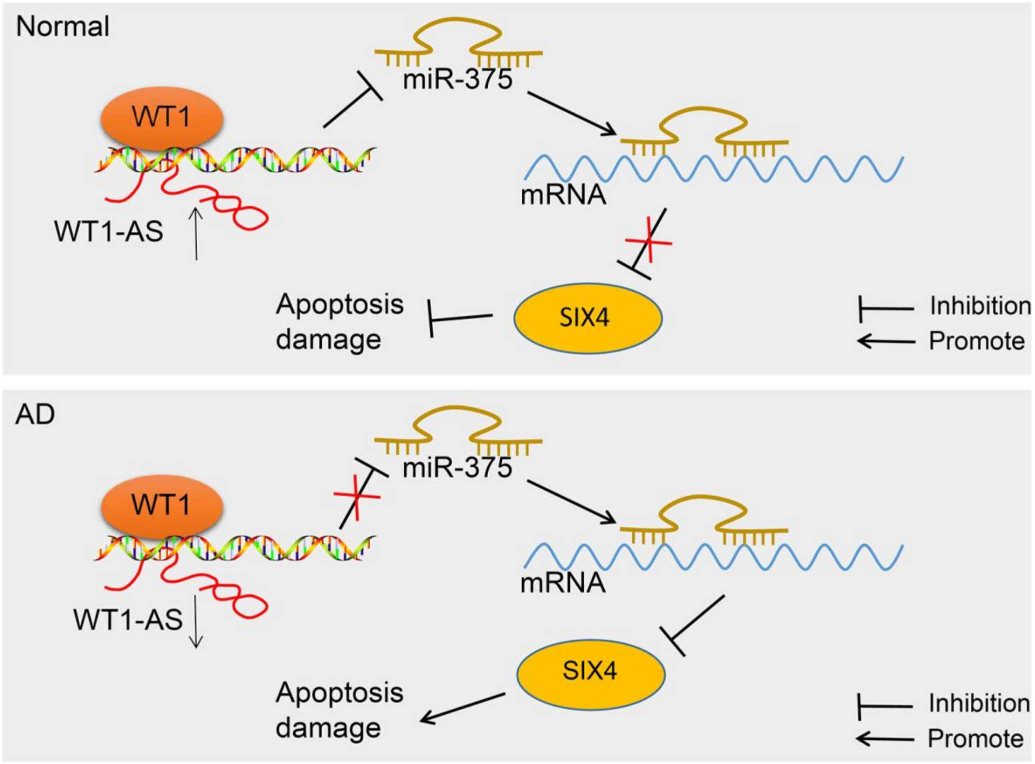

Supplementary Figure 1. WT1-AS was expressed at low levels in AD, and low expression of WT1-AS could promote the expression of the transcription factor WT1, thus promoting miR-375, inhibiting the expression of SIX4 and promoting oxidative stress injury and apoptosis of neurons in AD.
